# Collateral Damage: Neurological Correlates of Non-Fatal Overdose in the Era of Fentanyl-Xylazine

**DOI:** 10.1177/26331055241247156

**Published:** 2024-04-24

**Authors:** Dustin R Todaro, Nora D Volkow, Daniel D Langleben, Zhenhao Shi, Corinde E Wiers

**Affiliations:** 1 Center for Studies of Addiction, Department of Psychiatry, Perelman School of Medicine, University of Pennsylvania, Philadelphia, PA, USA; 2 Laboratory of Neuroimaging, National Institute on Alcohol Abuse and Alcoholism, National Institutes of Health, Bethesda, MD, USA; 3 Department of Radiology, Perelman School of Medicine, University of Pennsylvania, Philadelphia, PA, USA

**Keywords:** Addiction, fentanyl, heroin, xylazine, Hippocampus, hypoxia

## Abstract

Non-fatal opioid overdoses are associated with significant morbidity. Hypoxic brain injury caused by opioid-induced respiratory depression is a key mechanism of such morbidity. For example, reports describe an amnestic syndrome in opioid users associated with acute injury to the hippocampus, a brain region that is highly susceptible to hypoxic injury. In our recent study we investigated the effects of non-fatal opioid overdose on the hippocampal volume in a well-characterized sample of opioid use disorder (OUD) patients with a history of overdose (OD) compared to those with no prior overdose (NOD). Using structural magnetic resonance imaging (MRI) and voxel-based morphometry, we observed lower hippocampal volume in patients with a history OD than in the NOD group. These findings support an association between non-fatal opioid overdose and hippocampal injury, which we hypothesize contributes to recently reported cases of OUD related amnestic syndrome. Here we review our study findings and the potential pathophysiological mechanisms underlying the acute and delayed hippocampal injury in nonfatal opioid overdose. We also discuss the implications for the risk of overdose and brain injury with the increased prevalence of fentanyl and xylazine contamination of the illicit opioid supply. Lastly, we highlight considerations for clinical management of the underappreciated neurological injury and cognitive dysfunction in OUD patients.

**COMMENT ON:** Dustin R. Todaro, Xinyi Li, Laís da Silva Pereira-Rufino, Peter Manza, Ilya M. Nasrallah, Sandhitsu Das, Anna Rose Childress, Henry R. Kranzler, Nora D. Volkow, Daniel D. Langleben, Zhenhao Shi, Corinde E. Wiers, Hippocampal volume loss in individuals with a history of non-fatal opioid overdose, Addiction Biology, 28:e13336 (2023). https://doi.org/10.1111/adb.13336

Opioid use disorder (OUD) is a chronic, relapsing disease, accounting for up to 20% of all deaths among individuals 24 to 35 years of age in the US.^1^ With the increase in fatal ODs, there has also been a steady rise in the incidence of non-fatal ODs seen in the hospital and pre-hospital settings.^2^ Non-fatal opioid OD is associated with significant post-OD morbidity, including pulmonary edema, pneumonia, seizures, cardiac arrhythmia, cognitive deficits, depression, and increased risk of subsequent fatal overdose.^3-5^ However, available data on the neurological effects of non-fatal OD in humans are limited. Future studies on this population are needed to inform both short- and long-term treatment and reduce the risk of relapse, recurrent OD, and death.

Opioid OD is associated with profound respiratory depression and risk of hypoxic injury to susceptible brain regions, such as the hippocampus.^6^ Several case series/reports found impairment in verbal and spatial memory in opioid OD patients consistent with hippocampal injury, as recently discussed in the systematic review by Winstanley et al.^2^ Additionally, approximately 20 cases of opioid-associated amnestic syndrome in the United States and France have been attributed to acute hippocampal injury.^7^ Unfortunately, the history of OD in many of these cases is unavailable. Although fentanyl has been postulated to be the primary cause of opioid-associated amnestic syndrome (OAS), toxicology screening for novel synthetic opioids was not available in many of the case reports.^7^ While the direct neurotoxic effects of fentanyl and other unidentified contaminants may contribute to OAS, we hypothesize that non-fatal OD with opioid-induced respiratory depression and hypoxic brain injury is the common pathway to the hippocampal volume loss seen in many of these cases.

To systematically investigate the association between hippocampal volume and non-fatal opioid OD we employed a volumetric analysis of structural magnetic resonance imaging (MRI) data in OUD patients reporting at least one prior OD in their lifetime compared to those without OD (NOD).^8^ Opioid ODs were defined as loss of consciousness requiring a third party intervention to recover and included intentional suicide attempts.^8^ The cohorts were well characterized and showed no significant difference across demographic and clinical characteristics evaluated. For the OD group, the combined bilateral hippocampal volume was significantly lower compared to the NOD group (unadjusted mean ± SD = 0.48 ± 0.05 vs 0.51 ± 0.06, adjusted difference ± SE = 0.021 ± 0.010, *F*(1, 43) = 4.43, *P* = .041, partial η² = 0.09). Post hoc analysis of the left and right hippocampi separately identified a significant group difference only for the left hippocampus. The effect was robust under the regions-of-interest and voxel-wise analysis approaches and could not be accounted for by concurrent diagnosis of substance use disorders (ie, alcohol, stimulant, cannabis). Overall, our findings support the association between hippocampal injury and a history of non-fatal OD.

One important consideration with this study is that most OUD subjects (72.4%) were recruited between 2012 and 2014 prior to the near complete replacement of heroin with fentanyl in the national illicit opioid supply.^8-11^ The remainder of subjects were recruited between 2018 and 2022, a period marked by a significant increase in fentanyl-adulterated heroin in the Northeast and Midwest regions of the United States.^8,12,13^ Fentanyl is a synthetic opioid that is approximately 100 times more potent than morphine and is attributed to the significant rise in opioid ODs in the United States in the recent years.^10,14^ Several pharmacological properties of fentanyl contribute to the increased risk of OD with fentanyl intoxication. First, fentanyl’s high potency as a full mu opioid receptor agonist, narrow therapeutic window, and low safety index make relatively small fluctuations in the fentanyl content of illicit heroin potentially lethal.^10,15^ Second, fentanyl is highly lipophilic, which allows for a very fast brain uptake and almost immediate pharmacological effects.^14,16^ Consequently, there may be a reduced window of time to intervene with reversal agents such as naloxone during acute fentanyl overdose relative to other opioids.^16^ Preclinical models demonstrate prolongation of brain oxygen decreases with co-administration of heroin and fentanyl that is more pronounced than either drug alone.^17^ These observations are significant considering the ubiquity of fentanyl as an adulterant in the illicit heroin supply, underscoring the potential risk of OD and subsequent OD-related morbidity with concurrent fentanyl-heroin intoxication. We suspect that the brain structural changes identified in our study (ie, hippocampal volume loss) would likely be more pronounced in the setting of fentanyl-adulterated heroin OD. Additionally, the neurotoxic effects of fentanyl and other adulterants are not well known in humans and could further contribute to the short- and long-term neurological complications.

Xylazine (“tranq”), an α-2 receptor agonist used as a veterinary sedative and analgesic has recently emerged as an adulterant of the illicit fentanyl supplies. The presence of xylazine as an adulterant in opioid-related overdose deaths increased significantly in the United States between 2020 and 2022, with the highest prevalence in the Northeast, especially in Philadelphia, Pennsylvania.^18-20^ According to a 2021 Philadelphia Department of Public Health report, more than 90% of the illicit fentanyl supply in Philadelphia contained xylazine.^21^ As a central α-2 adrenergic receptor agonist, xylazine decreases release of norepinephrine and dopamine in the central nervous system, and at high doses may result in sedation, bradycardia, hypotension, and cardiac arrest.^22^ Xylazine taken in combination with fentanyl or other opioids may also potentiate adverse effects, thereby exacerbating hypoxemia and increasing risk for hypoxic brain injury.^22^ Interestingly, a recent multicenter, prospective cohort study looking at adult patients presenting to emergency departments (ED) with opioid overdose found that xylazine-positive patients were less likely to develop cardiac arrest or coma.^23^ Fentanyl was the most prevalent opioid detected among the study’s subjects.^23^ Several hypotheses are posited to explain this observation, including the possibility that patients consuming xylazine-adulterated opioids are exposed to a lower total opioid concentration, as some of the opioid is replaced with xylazine.^23^

Currently, the physiologic and biochemical mechanisms underlying xylazine toxicity in opioid-related overdose remain poorly understood. Recent work by Choi et al^24^ and Kiyatkin and Choi^25^ have begun to explore these mechanisms by examining the impact of xylazine on opioid-induced brain hypoxia in a preclinical rat model. Fentanyl (or heroin) administration is shown to induce biphasic changes in brain oxygen levels characterized by a rapid, transient decrease in oxygen followed by a weaker, post-hypoxic hyperoxia.^24,25^ This post-hypoxic “rebound” in oxygen levels appears in the brain but not the periphery and is thought to be a potential compensatory mechanism to counteract respiratory depression-induced brain hypoxia, possibly via CO_2_-mediated cerebral vasodilation and increased cerebral blood flow.^24,25^ Xylazine administration alone at large doses was shown to produce a weak, yet prolonged decrease in brain oxygen levels distinct from the profound, transient decreases seen with fentanyl and heroin.^24,25^ Coadministration of xylazine with fentanyl resulted in a more profound decrease in brain oxygen and elimination of the secondary increase in oxygen levels seen with fentanyl or heroin alone.^24,25^ These results suggest that xylazine adulteration of fentanyl (or heroin) may potentiate the risk of brain hypoxia with overdose.^24,25^ It is speculated that xylazine’s hypoxic effect may be independent of respiratory depression and result from cerebral vasoconstriction through α-2 receptor agonism on cerebral vessels.^24,25^ Further research will be required to reconcile these findings in preclinical models with recent clinical and translational studies.^23^ It is possible that the impact of xylazine on brain hypoxia in opioid-associated overdose is masked and/or underappreciated in studies using severe pathology such as cardiac arrest and/or coma as a primary outcome.^23^ Neuroimaging and cognitive studies in patients who have experienced xylazine-fentanyl/opioid overdose are needed to better understand the effects of xylazine on brain structure and function.

An additional complication associated with xylazine-adulterated fentanyl use is the development of severe necrotic skin ulcers.^26,27^ Skin ulcers have been reported to occur more often in individuals using xylazine compared to heroin alone and appear to be clinically unique relative to other injection drug use wounds.^28^ Xylazine skin wounds occur most frequently on extremities, and are often observed at locations distant to injection sites, even with intranasal or inhalation use.^26,27^ If left untreated, wounds are at risk of secondary bacterial infection and can lead to extensive limb necrosis.^26^ While preclinical studies have shown that xylazine exerts toxic effects on human umbilical vein endothelial cells in vitro by inhibiting cell proliferation and promoting apoptotic cell death, the underlying mechanism(s) of tissue injury in patients who use xylazine remains unclear.^29^ Consequently, there is a paucity of data to guide clinical treatment. These chronic, insidious wounds can also be a significant barrier to patients accessing care services, as inpatient and residential substance use treatment centers are often reticent to accept patients with ongoing necrotic wounds due to perceived medical complexity.^27^

A xylazine withdrawal syndrome has been described by patients and clinical practitioners, which further complicates clinical management and may have neurological consequences.^26^ Symptoms overlap those seen in opioid withdrawal and range from irritability/agitation, dysphoria, anxiety, body aches, tachycardia, and hypertension.^26^ Poorly controlled withdrawal is often cited as a causative factor for patient-directed discharge,^30,31^ ultimately placing patients at risk for progression of untreated necrotic skin wounds, sepsis, relapse, recurrent OD, and death. Overall, future studies are needed to define the long-term cognitive and neurologic effects of fentanyl-xylazine OD, along with the pathophysiology of xylazine-induced skin necrosis and withdrawal. Additionally, as more users shift from injecting to smoking fentanyl it becomes urgent to investigate the effects of fentanyl-xylazine combination to pulmonary tissue and lung function, as this could further jeopardize blood oxygenation.

While our study was not designed to explicate causal mechanisms of brain injury, we hypothesize that neurologic injury is multifactorial and reflects both acute and delayed pathological processes. Opioid-induced respiratory depression and hypoxemia are suspected to cause acute hippocampal injury and subsequent volume loss, as observed in our OD cohort. Additionally, models of traumatic brain injury (TBI) focused on post-TBI hippocampal atrophy suggest a progressive, neurodegenerative process may also lead to brain volume loss over time.^32^ These findings are consistent with studies identifying elevated levels of hyperphosphorylated tau protein in the hippocampus and entorhinal cortex in post-mortem brain tissue of opioid users.^33^ In Alzheimer’s disease, the accumulation of hyperphosphorylated tau in the hippocampus is associated with morphogenic changes and pre-mortem cognitive decline.^34^ Both preclinical and human studies have identified increased levels of hyperphosphorylated tau in response to hypoxic brain injury,^35^ supporting the hypothesis for a post-OD tauopathy potentially leading to progressive neurodegeneration. To better understand the effects of non-fatal opioid OD on hippocampal tau levels, future studies using positron emission tomography are needed.

Although our study focused on an a priori hippocampal region of interest, opioid OD may also impact the structural integrity of other brain regions. Indeed, exploratory whole-brain analysis of our data using a more liberal threshold for significance (voxel-level *P* < .005, cluster size > 600 mm^3^)^36^ suggested lower gray matter volume in several parietal and temporal regions among OD individuals (see Table 1). It is also worth noting from a functional connectivity perspective that the hippocampus serves as a critical hub for a diverse range of brain networks. Functional MRI studies have demonstrated dynamic reorganization of connectivity across distant brain structures in response to focal hippocampal lesions.^37^ For example, discrete hippocampal injury correlates with decreased functional connectivity between the thalamus and precuneus networks, which is thought to contribute to memory impairments seen in these patients.^37^ While acute hippocampal injury due to non-fatal overdose has been largely associated with cognitive deficits in memory function, a range of other neuropsychological deficits have been described in chronic OUD patients, including reduced abilities in cognitive flexibility, verbal working memory, verbal fluency, decision-making, attention, and processing speed.^38^ Moving forward, it will be important to disentangle the short- and long-term effects of OD on neurocognitive functioning to better inform care providers and address the evolving needs of patients.

**Table 1.** Exploratory whole-brain analysis of lower gray matter volume in OD than NOD individuals (voxel-level *P* < .005, cluster size > 600 mm^3^).

Region	Cluster size (mm^3^)	*Z*	MNI coordinates
Right superior parietal lobule	774	3.77	24.25/−59.25/65.75
Right temporal pole	1895	3.75	33.25/6.75/−47.25
Right superior parietal lobule	1182	3.74	32.25/−40.25/36.75
Left entorhinal area	900	3.41	−22.75/−4.25/−30.25
Left hippocampus	1141	3.26	−22.75/−14.25/−15.25
Left superior parietal lobule	669	3.23	−32.75/−50.25/36.75
Left supramarginal gyrus		3.12	−35.75/−41.25/39.75
Left temporal pole	1282	2.97	−37.75/19.75/−41.25

Abbreviation: MNI, montreal neurological institute.

Our recent study^8^ provides empirical evidence for underappreciated neurological injury in opioid OD patients. Despite observation that many OUD patients experience multiple opioid-related ODs, little is known about the impact this would have on a patient’s overall neurocognitive function. As neurological and cognitive impairments could interfere with a patient’s ability to engage in care and recover meaningfully, neurological evaluation and formal neuropsychological testing should be carefully considered as part of the acute overdose workup and ongoing substance use treatment, respectively. The potential for delayed and/or progressive worsening of cognitive function in non-fatal OD patients suggests a potential need for ongoing monitoring for cognitive impairment and neurorehabilitation in OUD treatment programs. Furthermore, the known risk for neuropsychiatric symptoms following TBI indicates that treatment for comorbid psychiatric conditions and/or behavioral disturbances should be central to any treatment plan.

## References

[1] Volkow ND Chandler RK Villani J. Need for comprehensive and timely data to address the opioid overdose epidemic without a blindfold. Addiction. 2022;117:2132-2134. 35611646 10.1111/add.15957

[2] Winstanley EL Mahoney JJ 3rd Castillo F Comer SD. Neurocognitive impairments and brain abnormalities resulting from opioid-related overdoses: a systematic review. Drug Alcohol Depend. 2021;226:108838. 34271512 10.1016/j.drugalcdep.2021.108838 PMC8889511

[3] Zibbell J Howard J Clarke SD Ferrell A Karon SL . Non-fatal Opioid Overdose and Associated Health Outcomes: Final Summary Report. Services USDoHaH, Evaluation OotASfPa, Office of Disability AaL-TCP; 2019. #HHSP233201600021I. Accessed March 4, 2019. https://aspe.hhs.gov/reports/non-fatal-opioid-overdose-associated-health-outcomes-final-summary-report-0#main-content

[bibr4-26331055241247156] Caudarella A Dong H Milloy MJ Kerr T Wood E Hayashi K. Non-fatal overdose as a risk factor for subsequent fatal overdose among people who inject drugs. Drug Alcohol Depend. 1 2016;162:51-55. 26993373 10.1016/j.drugalcdep.2016.02.024 PMC4833586

[5] Warner-Smith M Darke S Day C. Morbidity associated with non-fatal heroin overdose. Addiction. 2002;97:963-367. 10.1046/j.1360-0443.2002.00132.x 12144598

[6] Di Paola M Caltagirone C Fadda L Sabatini U Serra L Carlesimo GA . Hippocampal atrophy is the critical brain change in patients with hypoxic amnesia. Hippocampus. 2008;18:719-728. 18446831 10.1002/hipo.20432

[7] Barash JA Kofke WA. Connecting the dots: an association between opioids and acute hippocampal injury. Neurocase. 2018;24:124-131. 29774783 10.1080/13554794.2018.1475572

[8] Todaro DR Li X Pereira-Rufino LS , et al. Hippocampal volume loss in individuals with a history of non-fatal opioid overdose. Addict Biol. 2023;28:e13336. 10.1111/adb.13336 PMC10626561 37753562

[bibr9-26331055241247156] Ciccarone D. Fentanyl in the US heroin supply: a rapidly changing risk environment. Int J Drug Policy. 2017;46:107-111. 28735776 10.1016/j.drugpo.2017.06.010 PMC5742018

[bibr10-26331055241247156] Ciccarone D. The triple wave epidemic: supply and demand drivers of the US opioid overdose crisis. Int J Drug Policy. 2019;71:183-188. 30718120 10.1016/j.drugpo.2019.01.010 PMC6675668

[11] Mattson CL Tanz LJ Quinn K Kariisa M Patel P Davis NL. Trends and geographic patterns in drug and synthetic opioid overdose deaths - United States, 2013-2019. MMWR Morb Mortal Wkly Rep. 2021;70:202-207. 33571180 10.15585/mmwr.mm7006a4 PMC7877587

[12] Ciccarone D. The rise of illicit fentanyls, stimulants and the fourth wave of the opioid overdose crisis. Curr Opin Psychiatry. 2021;34:344-350. 33965972 10.1097/YCO.0000000000000717 PMC8154745

[13] O’Donnell J Gladden RM Goldberger BA Mattson CL Kariisa M. Notes from the field: opioid-involved overdose deaths with fentanyl or fentanyl analogs detected - 28 states and the district of Columbia, July 2016-December 2018. MMWR Morb Mortal Wkly Rep. 2020;69:271-273. 32163382 10.15585/mmwr.mm6910a4 PMC7075253

[14] Suzuki J El-Haddad S. A review: fentanyl and non-pharmaceutical fentanyls. Drug Alcohol Depend. 2017;171:107-116. 28068563 10.1016/j.drugalcdep.2016.11.033

[15] Yassen A Olofsen E Kan J Dahan A Danhof M. Pharmacokinetic-pharmacodynamic modeling of the effectiveness and safety of buprenorphine and fentanyl in rats. Pharm Res. 2008;25:183-193. 17914664 10.1007/s11095-007-9440-z PMC2190336

[16] Britch SC Walsh SL. Treatment of opioid overdose: current approaches and recent advances. Psychopharmacology (Berl). 2022;239:2063-2081. 35385972 10.1007/s00213-022-06125-5 PMC8986509

[17] Kiyatkin EA. Respiratory depression and brain hypoxia induced by opioid drugs: morphine, oxycodone, heroin, and fentanyl. Neuropharmacology. 2019;151:219-226. 30735692 10.1016/j.neuropharm.2019.02.008 PMC6500744

[18] Cano M Daniulaityte R Marsiglia F. Xylazine in overdose deaths and forensic drug reports in US States, 2019-2022. JAMA Netw Open. 2024;7:e2350630. 10.1001/jamanetworkopen.2023.50630 PMC10770774 38180756

[bibr19-26331055241247156] Friedman J Montero F Bourgois P , et al. Xylazine spreads across the US: a growing component of the increasingly synthetic and polysubstance overdose crisis. Drug Alcohol Depend. 2022;233:109380. 35247724 10.1016/j.drugalcdep.2022.109380 PMC9128597

[20] Kariisa M O’Donnell J Kumar S Mattson CL Goldberger BA. Illicitly manufactured fentanyl-involved overdose deaths with detected xylazine - United States, January 2019-June 2022. MMWR Morb Mortal Wkly Rep. 2023;72:721-727. 37384558 10.15585/mmwr.mm7226a4 PMC10328484

[21] Bettigole C Best A Da Silva DT. Xylazine (tranq) Exposure Among People Who Use Substances in Philadelphia. Health PDoP, Reduction DoSUPaH; 2022. PDPH-HAN-00417U-12-08-2022. Accessed December 8, 2022. https://hip.phila.gov/document/3154/PDPH-HAN_Update_13_Xylazine_12.08.2022.pdf/

[22] Ruiz-Colón K Chavez-Arias C Díaz-Alcalá JE Martínez MA. Xylazine intoxication in humans and its importance as an emerging adulterant in abused drugs: a comprehensive review of the literature. Forensic Sci Int. 2014;240:1-8. 24769343 10.1016/j.forsciint.2014.03.015

[23] Love JS Levine M Aldy K , et al. Opioid overdoses involving xylazine in emergency department patients: a multicenter study. Clin Toxicol (Phila). 2023;61:173-180. 37014353 10.1080/15563650.2022.2159427 PMC10074294

[24] Choi S Irwin MR Kiyatkin EA. Xylazine effects on opioid-induced brain hypoxia. Psychopharmacology (Berl). 2023;240:1561-1571. 37340247 10.1007/s00213-023-06390-y PMC10775769

[25] Kiyatkin EA Choi S. Brain oxygen responses induced by opioids: focus on heroin, fentanyl, and their adulterants. Front Psychiatry. 2024;15:1354722. 38299188 10.3389/fpsyt.2024.1354722 PMC10828032

[26] D’Orazio J Nelson L Perrone J Wightman R Haroz R. Xylazine adulteration of the heroin-fentanyl drug supply: a narrative review. Ann Intern Med. 2023;176:1370-1376. 37812779 10.7326/M23-2001

[27] Carroll JJ. Xylazine-associated wounds and related health concerns among people who use drugs: reports from front-line health workers in 7 US States. Subst Use Addctn J. 2024;45:222-231. 38258791 10.1177/29767342231214472

[28] Rodríguez N Vargas Vidot J Panelli J Colón H Ritchie B Yamamura Y. GC-MS confirmation of xylazine (Rompun), a veterinary sedative, in exchanged needles. Drug Alcohol Depend. 2008;96:290-293. 18472231 10.1016/j.drugalcdep.2008.03.005 PMC2527692

[29] Silva-Torres LA Vélez C Lyvia Alvarez J Ortiz JG Zayas B. Toxic effects of xylazine on endothelial cells in combination with cocaine and 6-monoacetylmorphine. Toxicol In Vitro. 2014;28:1312-1319. 25017475 10.1016/j.tiv.2014.06.013 PMC4181877

[30] Simon R Snow R Wakeman S. Understanding why patients with substance use disorders leave the hospital against medical advice: a qualitative study. Subst Abus. 2020;41:519-525. 31638862 10.1080/08897077.2019.1671942

[31] Thakrar AP Uritsky TJ Christopher C , et al. Safety and preliminary outcomes of short-acting opioid agonist treatment (sOAT) for hospitalized patients with opioid use disorder. Addict Sci Clin Pract. 2023;18:13. 36829242 10.1186/s13722-023-00368-z PMC9951406

[32] Bigler ED. Traumatic brain injury, neuroimaging, and neurodegeneration. Front Hum Neurosci. 2013;7:395. 23964217 10.3389/fnhum.2013.00395 PMC3734373

[33] Anthony IC Norrby KE Dingwall T , et al. Predisposition to accelerated Alzheimer-related changes in the brains of human immunodeficiency virus negative opiate abusers. Brain. 2010;133:3685-3698. 21126996 10.1093/brain/awq263

[34] Nelson PT Alafuzoff I Bigio EH , et al. Correlation of Alzheimer disease neuropathologic changes with cognitive status: a review of the literature. J Neuropathol Exp Neurol. 2012;71:362-381. 22487856 10.1097/NEN.0b013e31825018f7 PMC3560290

[35] Raz L Bhaskar K Weaver J , et al. Hypoxia promotes tau hyperphosphorylation with associated neuropathology in vascular dysfunction. Neurobiol Dis. 2019;126:124-136. 30010004 10.1016/j.nbd.2018.07.009 PMC6347559

[36] Lieberman MD Cunningham WA. Type I and Type II error concerns in fMRI research: re-balancing the scale. Soc Cogn Affect Neurosci. 2009;4:423-428. 20035017 10.1093/scan/nsp052 PMC2799956

[37] Henson RN Greve A Cooper E , et al. The effects of hippocampal lesions on MRI measures of structural and functional connectivity. Hippocampus. 2016;26:1447-1463. 27479794 10.1002/hipo.22621 PMC5082505

[38] Wollman SC Hauson AO Hall MG , et al. Neuropsychological functioning in opioid use disorder: a research synthesis and meta-analysis. Am J Drug Alcohol Abuse. 2019;45:11-25. 30359116 10.1080/00952990.2018.1517262

